# Comprehensive Analysis and Comparison of Amino Acid Levels in Cerebrospinal Fluid and Plasma of Children with Leukemia by the LC-MS Technique

**DOI:** 10.3390/ijms26051888

**Published:** 2025-02-22

**Authors:** Anna Kaliszewska, Piotr Struczyński, Tomasz Bączek, Maciej Niedźwiecki, Lucyna Konieczna

**Affiliations:** 1 Department of Pharmaceutical Chemistry, Medical University of Gdańsk, Hallera 107, 80-416 Gdańsk, Poland; anna.kaliszewska@gumed.edu.pl (A.K.); piotr.struczynski@gumed.edu.pl (P.S.); tbaczek@gumed.edu.pl (T.B.); 2 Department of Pediatrics, Hematology, Oncology and Endocrinology, Medical University of Gdańsk, Dębinki 7a, 80-211 Gdańsk, Poland; maciej.niedzwiecki@gumed.edu.pl

**Keywords:** amino acid, acute myeloid leukemia, acute lymphoblastic leukemia, plasma, cerebrospinal fluid, LC-MS

## Abstract

This study aimed to develop and optimize an analytical method for profiling 21 amino acids in cerebrospinal fluid and plasma, addressing the need for improved diagnostic tools in leukemia research. Using high-performance liquid chromatography coupled with electrospray ionization mass spectrometry, the method achieved enhanced resolution, sensitivity, and specificity. Rigorous sample preparation, including liquid–liquid extraction, ensured high recovery rates, while validation confirmed the method’s accuracy and reproducibility. Clinical application in pediatric leukemia patients revealed significant variations in amino acid concentrations across treatment stages, providing insights into disease progression and therapeutic response. Statistical analysis with IBM SPSS Statistics 25 compared amino acid levels in patients to healthy controls, identifying distinct patterns on day 1, day 15, and day 33 of treatment. Correlation analysis highlighted relationships between amino acid levels and factors such as treatment duration, sex, age, and blood test results. Key amino acids, including proline, leucine, and hydroxyproline, emerged as significant predictors of white blood cell count, effectively distinguishing between patient and control groups. This method demonstrates robust potential for broader leukemia research applications, pending further validation on larger cohorts.

## 1. Introduction

The analysis of amino acids in biological fluids plays a critical role in understanding various physiological and pathological conditions. Amino acids, being fundamental components of proteins and crucial metabolites, can provide valuable insights into the metabolic alterations associated with diseases, including cancer [1]. In particular, the evaluation of amino acid profiles in cerebrospinal fluid (CSF) and plasma can offer significant information for the diagnosis and monitoring of hematological malignancies such as leukemia [2,3].

In recent years, the determination of amino acid levels in plasma and cerebrospinal fluid has become increasingly sophisticated, driven by advancements in analytical techniques and their application in clinical research and diagnostics. Recent developments in HPLC, particularly coupled with fluorescence [4] or UV [5] detection, have improved the resolution and speed of amino acid separation and quantification. Newer HPLC methods, such as ion-exchange chromatography [6], provide detailed profiles of amino acid concentrations. Modern MS techniques, including tandem MS (MS/MS) [7], have significantly enhanced the sensitivity and specificity of amino acid analysis. These techniques enable precise quantification of amino acids and their metabolites in complex biological matrices like plasma and CSF. NMR spectroscopy is increasingly used for non-invasive and comprehensive amino acid profiling, offering insights into the metabolic state of patients and enabling the study of amino acid dynamics in biological fluids [8].

Leukemia, a malignant disorder of the blood and bone marrow, is often accompanied by profound metabolic changes [9]. These alterations can affect amino acid metabolism, making it essential to accurately profile amino acids to gain insights into disease progression [10]. Amino acid levels can also reflect treatment response. Studies have shown that specific amino acids in plasma and CSF correlate with disease and treatment efficacy, providing valuable insights for monitoring patients’ conditions [3]. Recent advancements in analytical techniques, particularly in liquid chromatography coupled with mass spectrometry (LC-MS), have significantly improved the sensitivity and accuracy of amino acid quantification in complex biological matrices [11].

This study focuses on the development and optimization of a robust analytical determination of 23 amino acids in CSF and plasma. Utilizing high-performance liquid chromatography coupled with electrospray ionization mass spectrometry (ESI-MS), the method aims to enhance the resolution, sensitivity, and specificity of amino acid detection. By refining chromatographic and mass spectrometric conditions, including the use of specific stationary phases, mobile phases, and optimized detector settings, the study seeks to achieve reliable quantification of amino acids in the context of leukemia.

Furthermore, the research involves rigorous method validation to ensure accuracy, specificity, and reproducibility. The study also explores various sample preparation techniques, including solid-phase and liquid–liquid extraction methods, to optimize recovery and minimize interference. The ultimate goal is to provide a validated analytical approach that can be applied to clinical studies, potentially aiding in the monitoring of disease status and therapeutic efficacy in pediatric leukemia patients.

The aim of this study was the development and validation of an effective analytical method for amino acid determination in biological fluids that would enhance our ability to investigate the metabolic profiles associated with leukemia, contributing to improved diagnostic and therapeutic strategies.

## 2. Results and Discussion

### 2.1. Optimization of Chromatographic and Mass Spectrometer Analysis Parameters

To optimize the chromatographic process and achieve the best separation of amino acids, the analysis was conducted using standard amino acid solutions, which were divided into three mixtures (MIX1, MIX2, and MIX3). The analysis was performed under various conditions, employing different linearly decreasing gradient elution programs for phase B, as well as varying column temperatures. Additionally, the effect of the mobile phase pH on the retention times of the amino acids was investigated. By using different buffers added to the phases, mobile phases with a broad pH range were obtained. The final stage of optimization involved examining how extending the analysis time would affect the separation of the amino acids.

Four pairs of amino acids with very similar retention times (Trp and Phe; Tyr and/or Pro and Val; Ala and Hyp; Gln/Asn and Ser) were the main focus during the optimization process. The final gradient elution program selected was 95–70% phase B due to its minimal separation of the Trp and Phe pair compared to other gradients. In the 95–60% phase B program, three amino acids showed nearly identical retention times; the 95–80% phase B program did not provide even minimal separation of pairs; the 95–90% phase B program failed to analyze all amino acids due to retention times exceeding 20 min.

Increasing the column temperature shortened the retention times of individual amino acids but did not enhance resolution. Consequently, a column temperature of 25 °C was chosen due to the low thermal stability of the analytes.

The use of mobile phases with different pH values did not significantly improve chromatographic separation. Instead, it led to extended retention times for individual analytes; thus, a mobile phase with pH = 3 was selected.

**Figure 1:**
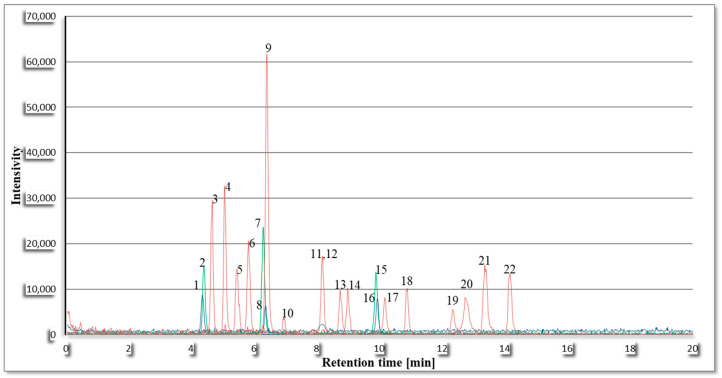
Chromatogram for shorter analysis time: 1—Tryptophan; 2—Phenylalanine; 3—Leucine; 4—Isoleucine; 5—Methionine; 6—Norvaline; 7—Valine; 8—Tyrosine; 9—Proline; 10—Cysteine; 11—Alanine; 12—Hydroxyproline; 13—Threonine; 14—Glycine; 15—Glutamine; 16—Serine; 17—Asparagine; 18—Glutamic acid; 19—Aspartic acid; 20—Histidine; 21—Arginine; 22—Lysine.

Extending the analysis time also did not yield satisfactory amino acid separation, so a shorter analysis time of 20 min was chosen. Figure 1 shows the chromatogram obtained using the shorter analysis time.

Due to the lack of complete chromatographic separation of up to 21 amino acids, it became necessary to utilize the Selected Ion Monitoring (SIM) function available in the mass spectrometer. Consequently, the amino acids were distributed among three SIM channels.

The final stage of optimizing the analytical method involved the optimization of mass spectrometer conditions. This optimization aimed to achieve the highest possible peak areas and heights, which could enhance the limits of quantification and detection, particularly for amino acids at low concentrations.

The collision energy (CE) was examined in the range of 50 to 200 V (Figures S1–S3). The optimal values were considered to be those within the range of 75–125 V (depending on the substance). Capillary voltage was examined in the range of 3 to 6 kV (Figures S4–S6). A value of 3000 V was selected as optimal.

### 2.2. Sample Preparation Procedure

#### 2.2.1. Solid-Phase Extraction

As a part of the conducted research, the optimal method for preparing samples for LC-MS analysis was selected. Initially, a solid-phase extraction method was developed. For this purpose, Strata-X-C 200 mg/3 mL cation-exchange columns were used. The essence of the extraction was that amino acids, under the influence of 0.1 M hydrochloric acid in water, were converted into cationic form, allowing them to interact with the column’s sorbent, which consisted of a porous silica gel material modified with chemical groups capable of the column’s filling, while the remaining compounds passed through the column’s bed. Subsequently, the compounds of interest were eluted using an appropriate solvent. However, this method did not meet the validation requirements for bioanalytical methods due to the low recovery of the SPE extraction, and it was found that the cause of the low recoveries was the significant loss of nine amino acids, which were eluted from the column bed during the washing step, which was an undesirable phenomenon. The remaining amino acids were eluted with an appropriate solution during the final, proper extraction step.

#### 2.2.2. Liquid–Liquid Extraction

Due to the unsatisfactory recovery results of amino acids during SPE, a new sample preparation method based on liquid–liquid extraction was developed. To obtain optimal extraction conditions, the effectiveness of deproteinization using various deproteinizing reagents was analyzed for both cerebrospinal fluid and plasma. Based on the signal intensity of the obtained amino acid peaks, the appropriate deproteinizing reagent for each biological material was selected: 0.01 M hydrochloric acids in water for cerebrospinal fluid and 0.1 M hydrochloric acid in methanol for plasma. The difference in reagent selection was due to the varying total protein content in the different biological fluids.

The developed sample preparation protocol allowed for effective extraction of the studied compounds from biological material. Table 1 shows the percentage recovery of the studied compounds for both sample preparation methods. The obtained values ranged from 93.55 to 101.58% for the cerebrospinal fluid samples and 93.29 to 100.55% for the plasma samples.

### 2.3. Method Validation

#### 2.3.1. Calibration Curve

Standard solutions for the calibration curves were prepared, and after conducting chromatographic analysis, the average calibration curve for each amino acid was determined. Among the 21 amino acids analyzed, it was not possible to determine the average calibration curve for cysteine. Cysteine was identified only qualitatively due to the inability to validate the calibration curve. This challenge is primarily attributed to its high reactivity and its tendency to oxidize to cystine during sample preparation. Table 2 presents the collected values of the regression coefficients (a and b), their standard deviations, the coefficient of determination, and the standard error of the correlation coefficient.

#### 2.3.2. Specificity

Specificity refers to the ability to accurately identify the analyte in the presence of other sample components, which may include auxiliary substances or contaminants. For a high level of specificity in an analytical method, it is essential to obtain an undistorted analytical signal from the analyte and to avoid interference. The specificity of the applied method was assessed by analyzing cerebrospinal fluid and plasma matrix samples obtained through solid-phase extraction (SPE) both with and without the addition of a mixture of amino acid standard solutions. No interference from the biological matrix with amino acid peaks was detected, which confirms the specificity of the proposed method.

#### 2.3.3. Linearity

Linearity refers to the change in the intensity of the analytical signal in relation to the corresponding change in the concentration of the analyte under investigation. The linearity of the applied method was determined by analyzing matrix samples of cerebrospinal fluid and plasma obtained through SPE, both with and without the addition of amino acid standard solution mixtures at different concentrations. The linearity was confirmed in a concentration range of 5 ng/mL–20 µg/mL.

#### 2.3.4. Limit of Detection and Limit of Quantification

The limit of detection (LOD) is related to a specific analytical procedure and is defined as the smallest amount or concentration of a substance that can be detected with a certain probability. It represents the minimum concentration of an analyte at which its presence in a sample can be reliably confirmed. In addition to being dependent on the analyte concentration level, the LOD also depends on other components of the sample.

The limit of quantification (LOQ) is defined as the smallest amount of concentration of a substance that can be quantitatively determined using the chosen analytical method with a specified level of accuracy and precision.

The LOD values ranged from 0.001 to 0.166 µg/mL, and the LOQ values ranged from 0.025 to 3 µg/mL.

Through the validation process described above, it has been demonstrated that the developed method for amino acid analysis in cerebrospinal fluid and plasma meets the requirements set for analytical procedures. The method in question is specific, rapid, straightforward, and can be successfully applied to amino acid analysis in diagnostic studies.

### 2.4. Results

The developed method was applied in a study conducted on a group of 60 children, from whom cerebrospinal fluid and plasma samples were collected. The cerebrospinal fluid samples were obtained as follows: from sick children (*n* = 37) at various stages of treatment—day 0 (*n* = 19), day 15 (*n* = 8), day 33 (*n* = 10)—experimental group; from children in the maintenance treatment stage, >33 days (*n* = 7)—control group. An identical division of samples was applied to the plasma: sick children at various stages of treatment (*n* = 21): day 0 (*n* = 8), day 15 (*n* = 5), day 33 (*n* = 8), and children in the maintenance treatment stage: >33 days (*n* = 6).

**Figure 2:**
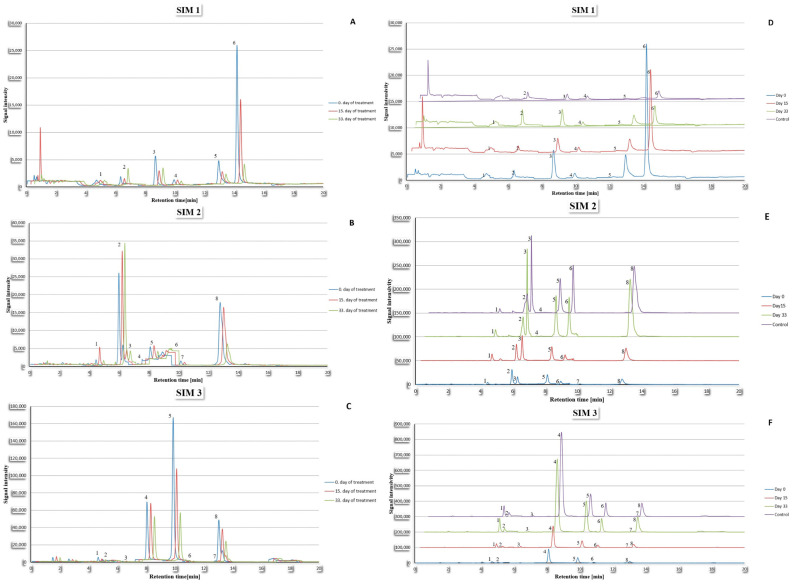
Chromatograms showing signals for SIM 1, 2, and 3 for cerebrospinal fluid sample of one randomly selected patient (**A**–**C**) and comparison of these signals with one randomly selected member of the control group (**D**–**F**). For SIM 1: 1—Tryptophan; 2—Proline; 3—Threonine; 4—Serine; 5—Aspartic acid; 6—Lysine. For SIM 2: 1—Phenylalanine; 2—Norvaline; 3—Valine; 4—Cysteine; 5—Alanine; 6—Glycine; 7—Asparagine; 8—Histidine. For SIM 3: 1—Leucine; 2—Isoleucine; 3—Tyrosine; 4—Hydroxyproline; 5—Glutamine; 6—Glutamic acid; 7—Arginine; 8—Homoarginine. The separation of the chromatograms in panels (**D**–**F**) was made to improve the visibility of the differences between them.

**Figure 3:**
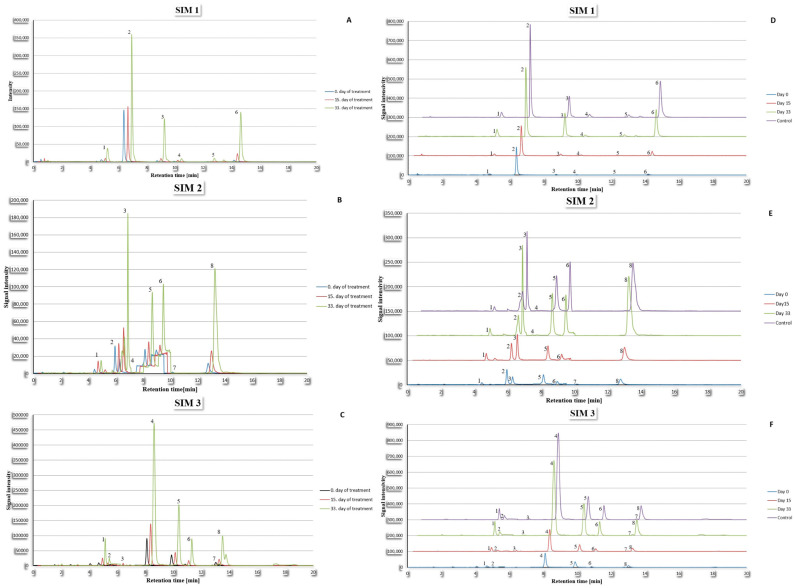
Chromatograms showing signals for SIM 1, 2, and 3 for plasma fluid sample of one randomly selected patient (**A**–**C**) and comparison of these signals with one randomly selected member of the control group (**D**–**F**). For SIM 1: 1—Tryptophan; 2—Proline; 3—Threonine; 4—Serine; 5—Aspartic acid; 6—Lysine. For SIM 2: 1—Phenylalanine; 2—Norvaline; 3—Valine; 4—Cysteine; 5—Alanine; 6—Glycine; 7—Asparagine; 8—Histidine. For SIM 3: 1—Leucine; 2—Isoleucine; 3—Tyrosine; 4—Hydroxyproline; 5—Glutamine; 6—Glutamic acid; 7—Arginine; 8—Homoarginine. The separation of the chromatograms in panels (**D**–**F**) was made to improve the visibility of the differences between them.

Figure 2 and Figure 3 display the chromatograms showing a comparison of amino acid concentration in cerebrospinal fluid and plasma for a randomly selected patient during their treatment, as well as comparison with a randomly selected volunteer from the control group.

**Figure 4:**
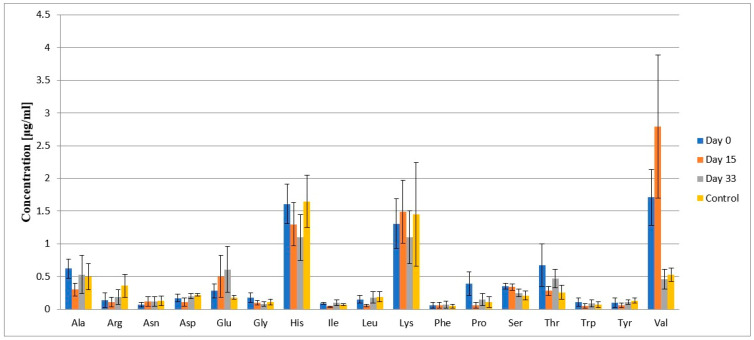
Mean concentrations of individual amino acids in cerebrospinal fluid and their standard deviations.

**Figure 5:**
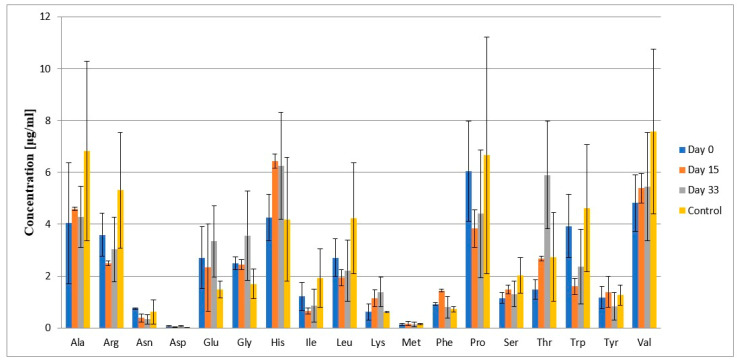
Mean concentrations of individual amino acids in plasma and their standard deviations.

Table 3, along with Figure 4, presents the mean concentrations of individual amino acids in cerebrospinal fluid and their standard deviations. Table 4 and Figure 5 show the mean concentrations of individual amino acids in plasma and their standard deviations. Changes in the concentration levels of specific amino acids can be observed depending on the treatment stage and in comparison with the control group.

Notably high changes in cerebrospinal fluid are seen for Gln and Hyp, with the highest concentrations at day 0 of treatment (the time of diagnosis), which then decrease with subsequent treatment stages, being the lowest in the control group. Similarly, His, Pro, and Thr have the highest mean concentrations at diagnosis. In contrast, the change in mean concentrations for Glu shows an opposite trend compared to Gln and Hyp, with its concentration increasing with further treatment. The mean concentration of Val is highest on day 15 of treatment, while the mean concentration levels of Arg on each day are lower than in the control group. The mean concentrations of other amino acids across different treatment days and in the control group are at similar levels. Met was not detected in the cerebrospinal fluid samples due to the too low concentration; Cys was present in the samples, but the mean concentration level was not calculated due to the lack of a calibration curve.

In the case of plasma, the mean concentrations of Gln and Hyp are highest on day 33 of treatment, which is the opposite to the concentration levels of these amino acids in cerebrospinal fluid. Similarly, the mean concentration of Gly, Glu, and Thr are highest on day 33. The mean concentration levels of Ala, Arg, and Val on different days are lower than in the control group. The mean concentrations of other amino acids across different treatment days and in the control groups are at similar levels. Met was detected in the plasma samples, but as with cerebrospinal fluid samples, the mean concentration level of Cys could not be calculated.

### 2.5. Statistical Analysis

The statistical analysis of amino acid levels in healthy individuals versus patients with acute myeloid leukemia and lymphoblastic leukemia was conducted using IBM SPSS Statistics 25 software (Endicott, NY, USA). A *p*-value of less than 0.05 was deemed statistically significant.

The Friedman test assessed differences across various time periods. Significant differences were found in cerebrospinal fluid samples for all amino acids except Lys (*p* > 0.05). In plasma samples, significant differences were observed for all amino acids.

The next step was the Spearman correlation analysis to determine if there is a statistically significant relationship between the studied variables. The analysis revealed statistically significant differences in the levels of specific amino acids between the beginning of the study and day 33. On day 33, there were statistically significantly lower levels of Gln, Trp, Pro, Thr, Leu, Phe, Ser, Hpro, Ile, and Gly in the cerebrospinal fluid. Conversely, statistically significantly higher levels on day 33 were observed for Asn, Tyr, Val, His, Asp, and Arg. In plasma samples, on day 33, there were statistically significantly lower levels of Gln, Pro, Leu, Phe, Gly, His, and Ala and higher levels of Trp, Asn, Tyr, Thr, Val, Asp, Arg, Ser, Hpro, and Lys. Statistically significant differences were also observed between the beginning of the study and day 15. For cerebrospinal fluid, there were statistically significantly lower levels of Gln, Trp, Pro, Thr, Leu, Phe, Ser, Hpro, Asp, Arg, Gly, Ala, and Ile and higher levels of Asn, Tyr, Val, and His. For plasma, there were statistically significantly lower levels of Gln, Pro, Val, Leu, Phe, Arg, Gly, Ala, Ile, and His and higher levels of Trp, Asn, Tyr, Thr, Ser, Hpro, Asp, and Lys. Statistically significant differences were also observed between days 15 and 33. For cerebrospinal fluid, there were statistically significantly lower levels of Gln, Pro, Thr, Hpro, Trp, and Ser and higher levels of Asn, Asp, Arg, Ala, and His. For plasma, there were statistically significantly lower levels of Gln, Pro, and Ala and higher levels of Asn, Val, Thr, Asp, and Arg.

Next, the analysis examined whether there is a statistically significant relationship between the passage of time and the levels of specific amino acids in cerebrospinal fluid. The correlation analysis revealed a negative relationship between time and the levels of Gln, Trp, Pro, Thr, Leu, Phe, Ser, Ile, Hpro, and Gly, indicating lower levels over time. Conversely, a positive correlation was observed for Asn, Tyr, Val, His, Asp, and Lys. There was no statistically significant relationship for Ala and Arg. The strongest correlation was found for Pro (Spearman’s ρ = −0.9, *p* < 0.0001). A similar analysis was conducted for plasma samples, revealing a negative relationship between the passage of time and levels of Gln, Pro, Leu, Phe, Ile, His, Gly, and Ala. Conversely, a positive correlation was observed for Trp, Asn, Tyr, Val, Thr, Ser, Hpro, Asp, and Lys. There was no statistically significant relationship for Arg. The strongest correlation was found for Gln (Spearman’s ρ = −0.94, *p* < 0.0001).

The Mann–Whitney U test was applied to compare two independent groups. In cerebrospinal fluid, 13 out of 18 amino acids did not differ from the control group on day 33 of treatment. Statistically significantly lower levels of amino acids in the control group were found for Gln, Trp (on days 0, 15, and 33), Pro, Hpro (on days 0 and 15), Phe, Ser (on day 0), and Ala (on day 15). Conversely, statistically significantly higher levels were observed for Asn (on days 0 and 15), Val, His (on day 0), Thr, Asp, and Arg (on days 0, 15, and 33). In plasma, fewer amino acids showed no statistically significant differences on day 33 compared to the control group (*n* = 13). On day 15 of treatment, only six amino acids showed no significant difference. Statistically significantly lower levels of amino acids in the control group were observed for Gln, Ala, and Pro (on days 0 and 15); Val, Leu, His, and Gly (on day 0); and Phe (on days 15 and 33). Conversely, statistically significantly higher levels were observed for Trp, Thr, Ser (on days 0, 15, and 33), Asn, Asp (on days 0 and 15), Tyr, Hpro (on day 0), Val (on days 15 and 33), Ile, and Arg (on day 15).

Based on the amino acid levels in plasma, five amino acids were identified that met both criteria: statistically significant differences between the compared groups before treatment and normalization of results, meaning no statistically significant differences on days 15 and 33 of treatment compared to the control group. These amino acids are Tyr, Leu, Hpro, His, and Gly.

The analysis also examined whether there is a statistically significant relationship between the levels of amino acids in plasma and cerebrospinal fluid. At the time of diagnosis, levels in plasma were strongly correlated with levels in cerebrospinal fluid, showing almost linear correlations, except for Trp, Asp, Ala, and Arg. Notable negative correlations included Leu in CSF and Phe in plasma; Phe in CSF and Leu in plasma; Gln in CSF and Ile in plasma; and Ile in CSF and Gln in plasma. In these cases, a lower level of one amino acid corresponded to a higher level of the other. Positive correlations included Ile in CSF and Hpro in plasma; Hpro in CSF and Ile and Arg in plasma; His in CSF and Lys in plasma; and Lys in CSF and His in plasma. On day 15 of treatment, similar strong correlations were observed (with exceptions for Asp, Ala, and Ar), showing almost linear correlations. Additionally, very strong correlations were observed. Notable correlations included Pro in CSF and Thr in Plasma; Thr in CSF and Pro in plasma; and Leu in CSF and Tyr in plasma. Among the negative correlations, notable pairs were Gln in CSF and Thr and Pro in plasma, and Ile in CSF and Trp in plasma. On day 33 of treatment, correlations remained strong with similar expectations. Notable positive correlations included Pro in CSF and Thr in plasma; Leu in CSF and Pro in plasma; and Leu in CSF and Asn in plasma. Notable negative correlations included Trp in CSF and Leu in plasma; Leu in CSF and Trp in plasma; and Asp in CSF and Tyr in plasma.

Further analysis examined whether the relationship between the passage of time and the levels of individual amino acids depends on the sex of the subjects. In cerebrospinal fluid samples, differences between girls (*n* = 15) and boys (*n* = 25) pertain to the relationship between time and the levels of Gly and Lys. In boys, a statistically significant relationship was found between time and Gly levels, while in girls, the relationship was with Lys levels. Statistically significant differences in plasma amino acid levels between girls and boys involved Gln and Lys at all time points, with girls having statistically significantly lower levels of these amino acids compared to boys, except for Lys on day 33, where girls had a significantly higher level. In plasma samples, differences between girls and boys pertain to the relationship between the passage of time and the levels of Phe, Ser, Ile, and Gly. Statistically significant relationships between the passage of time and the levels of these four amino acids were observed in boys. Similarly to the cerebrospinal fluid analysis, statistically significant differences in the levels of amino acids in plasma between girls and boys involve Gln and Lys across all time points. Girls had statistically significantly lower levels of these amino acids compared to boys, except for the level of Lys on day 33, where girls had a significantly higher level.

Subjects were divided into three age groups: up to 3 years (post-infancy period), up to 7 years (preschool age), and over 7 years (school age). For cerebrospinal fluid samples, the correlation analysis between the treatment period and the levels of individual amino acids showed statistically significant differences for most amino acids across the three age groups except for Asp, which did not correlate with the treatment period in the group of individuals older than 7 years. The same applies to Gly in those between 3 and 7 years. Noteworthy is the correlation between the treatment period and the level of Lys in the oldest group, as well as Ala levels in those up to 3 years of age. The Kruskal–Wallis test revealed two statistically significant differences in amino acid levels among the three age groups on day 33 of treatment: the level of Asn (χ^2^(2) = 9.21; *p* < 0.05) and the level of Hpro (χ^2^(2) = 7.19; *p* < 0.05). Individuals older than 7 years had a statistically significantly lower level (*p* < 0.05) of both amino acids compared to those aged up to 3 years. For plasma samples, statistically significant differences were noted for most amino acids across the three age groups—except for Lys, which did not correlate with treatment duration in the group of individuals up to 3 years old. Notable correlations between treatment duration and levels of Ser and Arg were observed in children older than 7 years, as well as Ile in those up to 3 years old. Kruskal–Wallis test analysis showed statistically significant differences in amino acid levels among the three age groups: before treatment, Hpro level (χ^2^(2) = 6.83; *p* < 0.05) and Arg level (χ^2^(2) = 8.9; *p* < 0.05); on the 15th day of treatment, Ala level (χ^2^(2) = 6.44; *p* < 0.05); on the 33rd day of treatment, Hpro level (χ^2^(2) = 7.19; *p* < 0.05). Individuals aged up to 3 years had a statistically significantly higher (*p* < 0.05) level of both amino acids compared to those aged 3 to 7 years. Individuals aged 3 to 7 years had a statistically significantly lower (*p* < 0.05) level of Ala in plasma compared to those aged over 7 years. Individuals up to 3 years had statistically higher (*p* < 0.05) levels of Hpro in plasma compared to those aged over 7 years.

The study examined the relationship between the laboratory blood test results and individual amino acids. Correlation analysis revealed several statistically significant negative correlations and one positive correlation between amino acid levels and the percentage of blasts in the blood and bone marrow. The strongest correlation was between His levels in cerebrospinal fluid and the percentage of blasts in the bone marrow; higher His levels corresponded to a lower percentage of blasts. Additionally, several statistically significant relationships were observed between amino acids and white blood cell count, platelets, and hemoglobin levels. The strongest were Trp in cerebrospinal fluid and Hpro in plasma, both negatively correlated with white blood cell count.

Numerous correlations were identified between amino acid levels and various CD markers. The strongest was between Pro levels in both biological materials and CD19; lower CD19 levels were associated with higher Pro concentrations. Further analysis on days 15 and 33 of treatment showed statistically significant correlations between amino acid levels on percentage of blasts. On day 15, a negative correlation was found between the percentage of blasts and Val levels in both plasma and cerebrospinal fluid. On day 33, statistically significant correlations were observed for Arg in cerebrospinal fluid and Ala in plasma. Unlike in cerebrospinal fluid, these are positive correlations. The Mann–Whitney U test analysis indicated that factors such as hepatomegaly or splenomegaly did not significantly affect amino acids levels in either plasma or cerebrospinal fluid (*p* < 0.05). However, there were statistically significant correlations between splenomegaly/hepatomegaly below the costal margin and amino acid levels: lower Trp in cerebrospinal fluid and higher Ala in plasma were associated with greater splenomegaly; the lower the Trp in cerebrospinal fluid, the greater the hepatomegaly. A statistically significant correlation observed was between the risk group and Arg levels in cerebrospinal fluid (ρ = 0.49; *p* < 0.01); higher Arg levels correspond to a higher risk group. The same applies to the biological material, which is plasma.

A model predicting white blood cell count based on cerebrospinal fluid amino acid levels was constructed with tree statistically significant predictors: Leu (Beta = 0.38; *p* < 0.01), Hpro (Beta = 0.73; *p* < 0.001), Asp (Beta = 0.25; *p* < 0.05). The strongest predictor of white blood cell count is the level of Hpro, followed by Leu. This model, which explained 54.7% of the variance in white blood cell count, was statistically significant: (F(3;36) = 16.67; *p* < 0.001). The higher the levels of these amino acids, the higher the white blood cell count. Below is the formula for the white blood cell count in this model: white blood cell count = Leu level × 90,170.12 + Hpro level × 435.79 + Asp level × 33,989.91 − 337,926.84. A model based on the level of amino acids in plasma confirms these results. Here, there are two statistically significant predictors: Hpro (Beta = 0.37; *p* < 0.01) and Leu (Beta = 0.7; *p* < 0.001). The strongest predictor of white blood cell count is the level of Hpro. This model, composed of these two predictors, is statistically significant (F(2;37) = 17.24; *p* < 0.001). It explains 45.4% of the variance in white blood cell count, which is less compared to the cerebrospinal fluid model. The higher the levels of these amino acids, the higher the white blood cell count. Other predictors, i.e., levels of other amino acids, are not statistically significant (*p* > 0.05).

Cluster analysis based on Hpro and Leu levels in plasma identified two clusters: individuals with high and low amino acid values. In the study group, 95% had high values (*n* = 38), while in the control group, only 35% (*n* = 7) did. The relationship between the two created clusters and the two compared groups is statistically significant (χ^2^(1) = 25.6; *p* < 0.001).

Regression analysis revealed that most correlations pertained to CD33. Two significant predictors in plasma were Pro (Beta = −0.52; *p* < 0.05) and His (Beta = −0.52; *p* < 0.05), explaining 38.3% of the variance in CD33 levels (F(2;14) = 5.96; *p* < 0.05). The lower the levels of these amino acids, the higher the level of CD33. In cerebrospinal fluid, Pro (Beta = −0.41; *p* < 0.05) and His (Beta = −0.6; *p* < 0.01) explained 46.1% of the variance (F(2;14) = 7.85; *p* < 0.01). The lower levels of these amino acids, the higher the level of CD33. Cluster analysis showed that all study group individuals (*n* = 40) had elevated amino acids levels in plasma, while only 5% (*n* = 1) of the control group did, a statistically significant relationship (χ^2^(1) = 55.61; *p* < 0.001).

**Figure 6:**
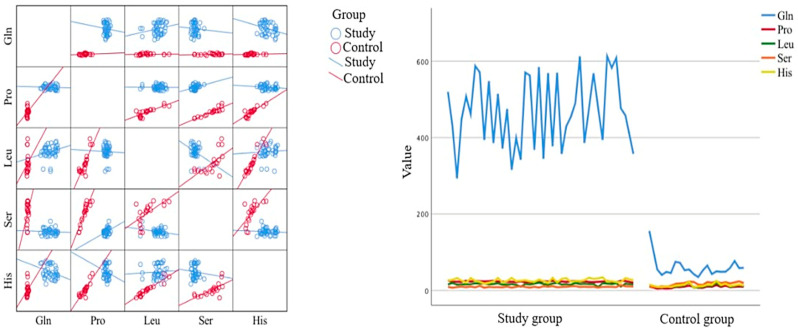
Visualization of statistical analysis of the results.

A regression analysis explained group variability (study vs. control group) by 99%. This model is statistically significant (F(5;54) = 940.67; *p* < 0.001). The statistically significant predictors in plasma are Gln (Beta = −0.15; *p* < 0.01), Pro (Beta = −0.66; *p* < 0.001), Leu (Beta = 0.06; *p* < 0.01), Ser (Beta = 0.23; *p* < 0.001), and His (Beta = −0.07; *p* < 0.05). The strongest predictor was Pro. Discriminant function analysis, analogous to multiple regression, confirmed these findings with a highly significant discriminant model (Wilks’ Lambda = 001, *p* < 0001), indicating a high proportion of explained variance (99%), making it the best discriminant model. Box’s M test indicated significant differences in group covariance matrices (F(15;5994.92) = 153.82; *p* < 0.001). The standardized coefficients for the discriminant functions supported the regression analysis results, showing statistically significantly lower levels of Gln, Leu, His, and Pro in the control group and higher levels of Ser (Figure 6).

The study described above conducted a detailed statistical analysis of amino acid levels in cerebrospinal fluid and plasma of patients with acute myeloid and lymphoblastic leukemia compared to healthy controls using various statistical methods. Significant differences in amino acid levels were observed over time, with distinct patterns identified between the beginning of the study and later time periods (days 15 and 33). Correlation analysis revealed both positive and negative relationships between amino acid levels and various factors, including treatment duration, sex, age, blood test results, and CD markers. Notably, the analysis identified key amino acids (e.g., Pro, Leu, Hpro) as significant predictors of white blood cell count and distinguished between study and control groups with high accuracy.

### 2.6. Discussion

Childhood leukemias are often diagnosed too late. The symptoms of acute leukemia in children are related to three main pathological processes: bone marrow failure due to extensive infiltration by malignant cells, metastasis to other tissues, and systemic effects caused by cytokines released from the pathological cells. Leukemia is first suspected when the child presents with classic symptoms of anemia; thrombocytopenia; and marked enlargement of the liver, spleen, or lymph nodes. However, the symptoms are often vague, nonspecific, and may mimic those of common childhood diseases [12]. Therefore, it is important to search for new compounds that may aid in earlier detection of this disease.

In cancer diseases such as leukemia, it is crucial to quickly and effectively determine the type of leukemia in order to apply the appropriate method of therapy, maximizing the chances of a complete cure. The basic test to detect the disease is the evaluation of the bone marrow and finding of more than 30% blasts in a microscopic examination of the bone marrow smear [13]. The final diagnosis is confirmed after the following: 1—Performing laboratory blood tests for anemia and thrombocytopenia; assessment of the leukocytes; and control of uric acid and dehydrogenase levels. 2—Conducting medical imaging tests such as chest X-rays. In cases involving the central nervous system, cerebrospinal fluid is examined. Currently, the diagnosis of CNS involvement is based on the presence of 5 leukocytes/mm^3^ in the cerebrospinal fluid along with the presence of blasts. The cerebrospinal fluid is collected from the patient by lumbar puncture [14], an invasive procedure often associated with complications, especially in children [12]. Therefore, it is desirable to explore the relationship between amino acid levels in the cerebrospinal fluid and plasma.

This paper presents a novel, optimized, and validated method for analyzing amino acid content in biological materials using the LC-MS technique. This method involves sample preparation by protein precipitation with hydrochloric acid in an appropriate solvent, bypassing the need for analyte derivatization. The proposed protocol enables both qualitative and quantitative analysis of 21 amino acids and two internal standards. Our method, which achieves limits of detection between 0.001 and 0.166 µg/mL and limits of quantification between 0.025 and 3 µg/mL across a linear dynamic range of 5 ng/mL to 20 µg/mL with recovery rates consistently between 93% and 102%, represents a significant improvement over classical approaches. The use of LC-MS resulted in lower limits of detection and quantification compared to fluorescence detection methods [4]. Additionally, this study achieved a lower detection limit range compared to the analysis presented by Virág et al. [7], which used LC-MS/MS with sample preparation based on deproteinization with trifluoroacetic acid and achieved an LLOQ between 0.5 and 1 µg/mL. Other earlier studies, such as that by Peng et al. [15], employed ion-exchange chromatography with post-column ninhydrin derivatization and typically reported detection limits around 1 µM. In contrast, our optimized liquid–liquid extraction protocol combined with Selected Ion Monitoring (SIM) enables the reliable detection of low-abundance analytes in the limited CSF volumes (50 µL) available from pediatric patients.

Our findings also complement and extend those from recent global metabolomics studies. The study by Brown et al. [3] demonstrated that comprehensive CSF profiling in pediatric acute lymphoblastic leukemia (ALL) can identify metabolites associated with cancer-related fatigue (CRF). In that work, gamma-glutamylglutamine and asparagine were found to be significantly associated with CRF—with combined *p*-values of 6.2 × 10^−6^ and 3.5 × 10^−4^, respectively—suggesting a role for glutamatergic neurotransmission and oxidative stress in the pathophysiology of CRF. Our targeted quantification not only confirms these associations but also provides the sensitivity necessary for precise monitoring of these key metabolites, potentially facilitating their translation into clinically useful biomarkers.

Furthermore, the results reported by Protas et al. [16] indicate that excitatory amino acids (EAAs) such as glutamate and aspartate are significantly elevated in CSF at critical treatment phases—specifically, after induction of remission and during the consolidation phase. These increased levels, observed in the absence of elevated total protein or albumin quotients and independent of age and gender, suggest that the elevations in EAAs reflect underlying neuronal abnormalities rather than a disruption of the blood–brain barrier. Although our study did not observe overt clinical neurotoxicity, the persistent elevation of these amino acids may signal subclinical neurotoxicity induced by intensive chemotherapy regimens that include agents such as methotrexate, prednisone, and L-asparaginase.

Method optimization also allowed for the elimination of the time-consuming derivatization procedure used in other publications [4,17]. It is important to highlight that research involving pediatric populations poses significant challenges due to the limited availability of biological material, with the collection of CSF being particularly invasive and associated with risks of complications.

Despite the limited number of samples, our results revealed statistically significant differences in amino acid levels in both CSF and plasma between patients and the control group. The identification of such changes indicates the potential diagnostic and prognostic values of amino acid profiling. In future research phases, we plan to increase the sample size, which will allow for even more reliable findings and enable the development of comprehensive analyses, including predictive models to better forecast patients’ hematological status and monitor treatment responses.

## 3. Materials and Methods

### 3.1. Chemicals

Analytical standards of 23 amino acids (alanine—Ala; arginine—Arg; asparagine—Asn; cysteine—Cys; phenylalanine—Phe; glycine—Gly; glutamine—Gln; histidine—His; homoarginine—Harg; hydroxyproline—Hyp; Isoleucine—Ile; aspartic acid—Asp; glutamic acid—Glu; leucine—Leu; methionine—Met; norvaline—Nva; proline—Pro; serine—Ser; threonine—Thr; tryptophan—Trp; tyrosine—Tyr; valine—Val) were purchased from Biochemika Fluka Chemie GmbH (Buchs, Switzerland). Ammonia, formic acid, and ammonium formate were obtained from Sigma-Aldrich (St. Louis, MO, USA). Acetonitrile and methanol were acquired from J.T. Baker (Deventer, The Netherlands). Hydrochloric acid was sourced from Polskie Odczynniki Chemiczne (Gliwice, Poland). Ultrapure water was obtained using a Milli-Q purification system (Millipore, Bedford, NY, USA).

### 3.2. Instrumentation

The HPLC system used for the determination was the Agilent Technologies 1260 Infinity (Agilent Technologies, Santa Clara, CA, USA), comprising an autosampler (1260 Autosampler G1329A, Agilent Technologies, Santa Clara, CA, USA), a degasser for the mobile phase (1260 Degasser G1322A, Agilent Technologies, Santa Clara, CA, USA), a UV–VIS detector (1260 VWD G1314F, Agilent Technologies, Santa Clara, CA, USA), a chromatographic column—Xbridge Amide^TM^ (3.0 × 100 mm; 3.5 µm) (Waters, Miliford, MA, USA), a binary pump (1260 Bin Pump G1312B, Agilent Technologies, Santa Clara, CA, USA), and a column thermostat (1290 Thermostat G1330B, Agilent Technologies, Santa Clara, CA, USA). The HPLC chromatographic system was used in conjunction with a mass spectrometer equipped with an electrospray ionization (ESI-MS) source and a quadrupole analyzer (6120 Quadrupole LC-MS G6120B, Agilent Technologies, Santa Clara, CA, USA). Chemstation Rev. B.04.02 SP1 software (Agilent Technologies, Santa Clara, CA, USA) was used for data analysis.

Strata-X-C 200 mg/3 mL SPE columns were obtained from Phenomenex (Torrance, CA, USA). A T460IH ultrasonic bath was purchased from Ultron (Dywity, Poland). Automatic pipettes were sourced from Biohit (Helsinki, Finland). The CentriVap vacuum concentrator was bought from Labconco Corporation (Kansas City, MO, USA). AS 110/C/2 analytical balance was obtained from Radwag (Radom, Poland). MPW-250 and MPW-211 laboratory centrifuges were purchased from Danlab (Białystok, Poland). Yellowline TTS2 single point shaker was sourced from IKA Works Inc. (Wilmington, NC, USA). VACElut SPS 24 liquid–solid-phase vacuum extraction set was bought from Agilent Technologies (Santa Clara, CA, USA).

### 3.3. Preparation of Standard Solutions

Standard solutions of each amino acid were prepared by dissolving 10 mg of the analyte in 10 mL deionized water (for tyrosine, a mixture of deionized water and formic acid (8:2, *v*/*v*) was necessary due to its insolubility in water). These solutions were then mixed and diluted with acetonitrile to obtain MIX solutions with concentrations of 100 ng/mL, 1 µg/mL, 10 µg/mL, and 100 µg/mL with the following composition: MIX1—Ala, His, Ile, Lys, Pro, Ser, Thr, and Val; MIX2—Hyp and Gln; MIX3—Arg, Asn, Cys, Phe, Gly, Asp, Glu, Met, Trp, and Tyr. Two internal standard solutions (Harg and Nva) were prepared at a concentration of 1 mg/mL using the same method.

### 3.4. Sample Collection

The developed analytical method was employed to determine the concentrations of endogenous amino acids in cerebrospinal fluid and plasma from a group of children with leukemia as well as a control group of children. The research was conducted with the Department of Clinic of Pediatrics, Hematology, and Oncology at the Medical University of Gdansk, following approval from the Independent Bioethics Committee for Scientific Research at the Medical University of Gdańsk. This study was conducted in compliance with the Declaration of Helsinki, and approved by the Institutional Review Board (or Ethics Committee) of Medical University of Gdańsk (protocol code NKEBN/4-58/2012 2012-02-18). The study involved 60 children from whom cerebrospinal fluid and/or plasma samples were collected. A total of 44 cerebrospinal fluid samples were analyzed, including 37 from children with acute myeloid or lymphoblastic leukemia at various stages of treatment (0, 15, and 33 days of treatment). The control group consisted of 7 samples from children undergoing maintenance therapy (>33 days). An identical distribution was applied to the plasma samples, with 27 samples analyzed. We acknowledge that the sample size is a limitation of this study. However, due to the challenges associated with pediatric research and the invasive nature of cerebrospinal fluid collection, this pilot study focused on optimizing the analytical method and identifying preliminary metabolic patterns, which will serve as a foundation for future research with larger cohorts. The cerebrospinal fluid and blood samples were collected, with blood samples additionally centrifuged to remove cellular components, and were then frozen immediately after collection and stored at −80 °C.

### 3.5. Sample Preparation

A total 6 µL of norvaline standard solution with a concentration of 0.5 mg/mL and 15 µL of homoarginine standard solution at a concentration of 10 µg/mL were added to 50 µL of cerebrospinal fluid or plasma sample. Subsequently, 79 µL of a protein precipitation reagent was added to the sample: 0.1 M hydrochloric acid in water for cerebrospinal fluid or 0.1 M hydrochloric acid in methanol for plasma. The samples were then centrifuged at 10,000 rpm for 8 min. An aliquot of 100 µL was taken and evaporated to dryness at 45 °C for 1 h. The dry residue was dissolved in 150 µL of an acetonitrile−water mixture (8:2, *v*/*v*) and then centrifuges again at 10,000 rpm for 8 min. The prepared sample was then subjected to LC-MS analysis.

### 3.6. Final Chromatographic Analysis Parameters

The UV spectrophotometer detector was set to 254 nm. The mobile phase consisted of 10 mM ammonium formate with pH 3 in water (phase A) and 10 mM ammonium formate in acetonitrile (phase B). The gradient elution program was as follows: at 0 min—95% Phase B; at 20 min—70% phase B; at 20.1 min—95% phase B; at 30 min—95% phase B. The flow rate of the mobile was 1 mL·min. The used column was Xbridge AmineTM (3.0 × 100 mm; 3.5 um), and it was thermostated at 25 °C. The sample volume injected was 10 uL.

The mass spectrometer working conditions were as follows: fragmentation voltage in the range of 75–125 V (depending on the analyte), ionization method—electrospray, voltage applied to the capillary—3000 V, gas flow (nitrogen)—12 L/min, temperature of the nebulizing gas—250 °C.

## 4. Conclusions

The LC-MS method developed was optimized to allow for simple sample preparation while ensuring sensitive, selective, accurate, and precise quantification of an amino acid panel. This optimization enabled the method to be successfully used in a wide variety of studies, where it proved to be highly robust and consistently yielded reliable results. Furthermore, while the method can be used for diagnostic purposes and metabolomics studies, it is also suitable for monitoring treatment effects. It should be emphasized that the small sample volume required for analysis using this methodology of measuring amino acid levels in CSF and plasma of pediatric patients holds potential for detecting biomarkers of acute lymphoblastic leukemia. Moreover, a relationship between CSF and plasma amino acid levels was observed. Our findings suggest that amino acid profiles may serve as valuable biomarkers for monitoring leukemia progression and treatment response. For instance, leucine, hydroxyproline, and aspartic acid demonstrated significant associations with white blood cell counts, which may assist in predicting hematological parameters. In the future, we see the potential for such measurements to be employed as an additional diagnostic tool supporting personalized therapy.

## Figures and Tables

**Table 1:** Values of recovery for analyzed amino acids measured using CSF and plasma.

Amino Acid	Recovery [%]	
	Cerebrospinal Fluid	Plasma
Alanine	96.65 (94.7–98.5)	95.36 (93.2–98.1)
Arginine	95.22 (93.26–97.2)	94.43 (92.3–96.5)
Asparagine	93.78 (91.7–95.66)	94.22 (92.5–96.1)
Cysteine	95.47 (93.5–97.74)	93.29 (91.43–95.3)
Phenylalanine	98.62 (96.91–100.28)	99.36 (97.47–101.25)
Glycine	97.55 (95.83–99.34)	95.24 (93.19–96.98)
Glutamine	99.96 (98.12–101.7)	100.25 (98.32–102.18)
Histidine	98.88 (96.97–100.45)	96.33 (94.6–98.33)
Homoarginine	97.66 (95.36–99.56)	97.25 (95.76–98.97)
Hydroxyproline	100.23 (98.56–101.88)	99.99 (98.13–102.23)
Isoleucine	101.58 (99.14–103.78)	100.56 (98.57–102.31)
Aspartic acid	96.33 (95.02–98.21)	95.37 (93.71–97.13)
Glutamic acid	97.26 (95.2–99.11)	98.21 (96.5–99.91)
Leucine	96.28 (94.67–98.15)	95.14 (93.59–96.83)
Lysine	94.55 (92.66–96.32)	96.32 (94.21–98.38)
Methionine	93.12 (92.01–95.23)	95.25 (93.49–97.2)
Norvaline	99.52 (97.62–101.6)	100.01 (98.39–101.75)
Proline	100.02 (98.63–101.81)	99.83 (96.93–101.2)
Serine	93.55 (92.13–95.17)	95.18 (93.48–97.08)
Threonine	96.64 (94.9–98.53)	98.21 (96.33–100.05)
Tryptophan	95.19 (93.17–97.14)	95.14 (94.03–96.15)
Tyrosine	94.49 (93.17–95.88)	95.98 (94.12–97.98)
Valine	98.36 (96.8–99.92)	98.74 (96.64–100.34)

**Table 2:** Calibration curves for analyzed amino acids in CSF samples.

Amino Acid	Calibration Curve y = ax + b				R^2^ *	*s* **
	a	(±SD) ^1^	b	(±SD) ^2^		
Alanine	0.23768	0.000221	0.00432	0.017908	0.999483	0.041639
Arginine	0.033134	0.016347	0.010492	0.132593	0.998403	0.308293
Asparagine	0.130382	0.000459	−0.00026	0.003327	0.999901	0.009052
Phenylalanine	0.294056	0.002133	0.017366	0.016311	0.999632	0.041272
Glycine	0.148241	0.001787	−0.00204	0.016742	0.999419	0.030993
Glutamine	0.020109	0.000104	−0.00114	0.003187	0.999814	0.007881
Histidine	0.26369	0.000219	0.001032	0.020489	0.999725	0.037931
Hydroxyproline	0.037831	0.000274	0.001557	0.00799	0.999582	0.021309
Isoleucine	0.494622	0.001565	−0.00228	0.011351	0.99992	0.030882
Aspartic acid	0.106095	0.000313	0.029904	0.00239	0.999939	0.006048
Glutamic acid	0.173118	0.000746	−0.00321	0.005701	0.99987	0.014426
Leucine	0.37182	0.000214	0.021159	0.017393	0.9998	0.040442
Lysine	0.236996	0.001738	−0.008	0.014094	0.999678	0.032769
Methionine	0.277166	0.000898	−0.00291	0.00687	0.999926	0.017384
Proline	1,120,977	0.004009	−0.00327	0.029084	0.999898	0.079128
Serine	0.087556	0.001281	0.048316	0.009297	0.998289	0.025293
Threonine	0.113684	0.000758	0.044726	0.0058	0.999689	0.014677
Tryptophan	0.211539	0.001083	−0.00296	0.008282	0.9998	0.020957
Tyrosine	0.066164	0.000435	0.003175	0.003326	0.999698	0.008415
Valine	0.297504	0.002691	−0.00091	0.020578	0.999428	0.052068

R^2^ *—coefficient of determination; s **—standard error of the correlation coefficient; (±SD) ^1^—standard deviation of the regression coefficient a; (±SD) ^2^—standard deviation of the regression coefficient b.

**Table 3:** Comparison of amino acid levels in CSF samples collected from leukemia patients and subjects from the control group.

Amino Acid	Patients During Treatment						Control Group	
	Day 0		Day 15		Day 33			
	Average Concentration [μg/mL]	±SD **	Average Concentration [μg/mL]	±SD **	Average Concentration [μg/mL]	±SD **	Average Concentration [μg/mL]	±SD **
Ala	0.62	±0.15	0.3	±0.10	0.53	±0.28	0.49	±0.20
Arg	0.14	±0.11	0.11	±0.07	0.19	±0.11	0.36	±0.17
Asn	0.07	±0.03	0.12	±0.07	0.12	±0.07	0.13	±0.07
Asp	0.17	±0.06	0.11	±0.06	0.2	±0.04	0.22	±0.02
Gln	456.93	±97.11	256.29	±17.15	139.01	±11.78	80.26	±12.8
Glu	0.28	±0.11	0.5	±0.32	0.61	±0.35	0.18	±0.03
Gly	0.18	±0.07	0.1	±0.03	0.08	±0.03	0.11	±0.04
His	1.61	±0.30	1.3	±0.33	1.09	±0.34	1.65	±0.40
Hpro	391.5	±75.67	103.71	±25.22	64.11	±11.81	50.19	±11.70
Ile	0.09	±0.01	0.05	±0.01	0.1	±0.04	0.07	±0.01
Leu	0.15	±0.06	0.06	±0.02	0.18	±0.09	0.19	±0.08
Lys	1.31	±0.37	1.49	±0.48	1.1	±0.40	1.45	±0.79
Phe	0.06	±0.04	0.06	±0.04	0.07	±0.05	0.05	±0.03
Pro	0.39	±0.18	0.06	±0.04	0.15	±0.09	0.11	±0.08
Ser	0.35	±0.04	0.34	±0.05	0.25	±0.06	0.21	±0.07
Thr	0.67	±0.32	0.28	±0.07	0.47	±0.14	0.26	±0.10
Trp	0.11	±0.06	0.05	±0.03	0.09	±0.05	0.07	±0.04
Tyr	0.1	±0.07	0.06	±0.03	0.11	±0.03	0.13	±0.04
Val	1.71	±0.43	2.79	±1.09	0.46	±0.15	0.53	±0.10

** ±SD—standard deviation.

**Table 4:** Comparison of amino acid levels in plasma samples collected from leukemia patients and subjects from the control group.

Amino Acid	Patients During Treatment						Control Group	
	Day 0		Day 15		Day 33			
	Average Concentration [μg/mL]	±SD **	Average Concentration [μg/mL]	±SD **	Average Concentration [μg/mL]	±SD **	Average Concentration [μg/mL]	±SD **
Ala	4.05	±2.34	4.6	±0.07	4.29	±1.17	6.83	±3.47
Arg	3.59	±0.83	2.49	±0.09	3.03	±1.25	5.31	±2.23
Asn	0.75	±0.04	0.39	±0.15	0.33	±0.19	0.62	±0.46
Asp	0.06	±0.03	0.03	±0.01	0.07	±0.02	0.01	±0.008
Gln	47.22	±23.68	95.87	±28.83	129.03	±34.84	40.03	±15.52
Glu	2.71	±1.19	2.33	±1.69	3.34	±1.37	1.48	±0.33
Gly	2.49	±0.24	2.44	±0.19	3.56	±1.73	1.7	±0.57
His	4.26	±0.90	6.43	±0.27	6.26	±2.05	4.19	±2.38
Hpro	76.18	±10.68	142.79	±21.06	248.24	±70.74	33.64	±15.29
Ile	1.22	±0.54	0.65	±0.13	0.86	±0.63	1.92	±1.13
Leu	2.71	±0.73	1.95	±0.31	2.21	±1.18	4.24	±2.13
Lys	0.612	±0.31	1.15	±0.32	1.38	±0.57	0.6	±0.03
Met	0.13	±0.03	0.17	±0.08	0.14	±0.08	0.16	±0.03
Phe	0.92	±0.05	1.44	±0.05	0.8	±0.42	0.72	±0.11
Pro	6.05	±1.93	3.84	±0.72	4.4	±2.46	6.66	±4.56
Ser	1.15	±0.21	1.49	±0.18	1.31	±0.50	2.03	±0.69
Thr	1.49	±0.37	2.69	±0.09	5.9	±2.07	2.74	±1.71
Trp	3.93	±1.22	1.61	±0.32	2.36	±1.43	4.63	±2.45
Tyr	1.17	±0.43	1.39	±0.59	0.83	±0.53	1.27	±0.39
Val	4.82	±1.09	5.39	±0.57	5.46	±2.08	7.58	±3.182

** ±SD—standard deviation.

## Data Availability

Data will be made available on request.

## Supplementary Materials

The following supporting information can be downloaded at https://www.mdpi.com/article/10.3390/ijms26051888/s1.

## Abbreviations

The following abbreviations are used in this manuscript:

CSF	Cerebrospinal fluid
ESI-MS	Electrospray ionization mass spectrometry
SIM	Selected Ion Monitoring
CE	Collision energy
SPE	Solid-phase extraction
LOD	Limit of detection
LOQ	Limit of quantification
