# Supplementary material for: Comprehensive Analysis and Comparison of Amino Acid Levels in Cerebrospinal Fluid and Plasma of Children with Leukemia by the LC-MS Technique
Source: Int J Mol Sci. 2025 Feb 22;26(5):1888. doi: 10.3390/ijms26051888 (PMC11899944; doi:10.3390/ijms26051888)
Supplement: Supplementary file 1 [file ijms-26-01888-s001.zip › ijms-3428254-supplementary.pdf]

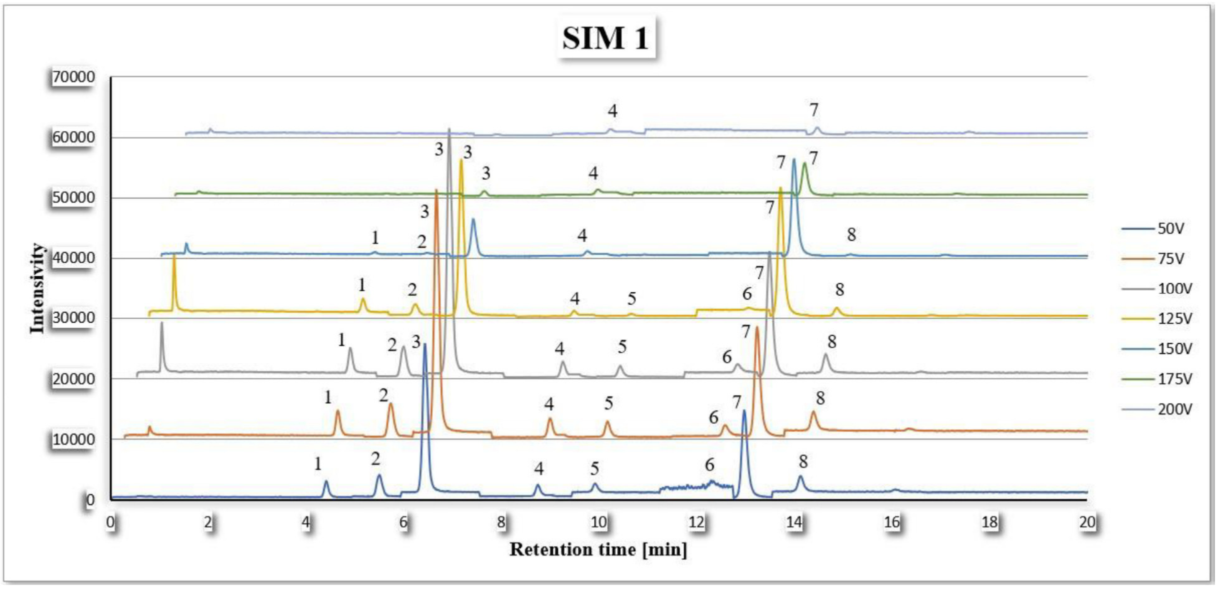

Figure S1. Chromatogram of signal intensity for different CE for SIM 1.

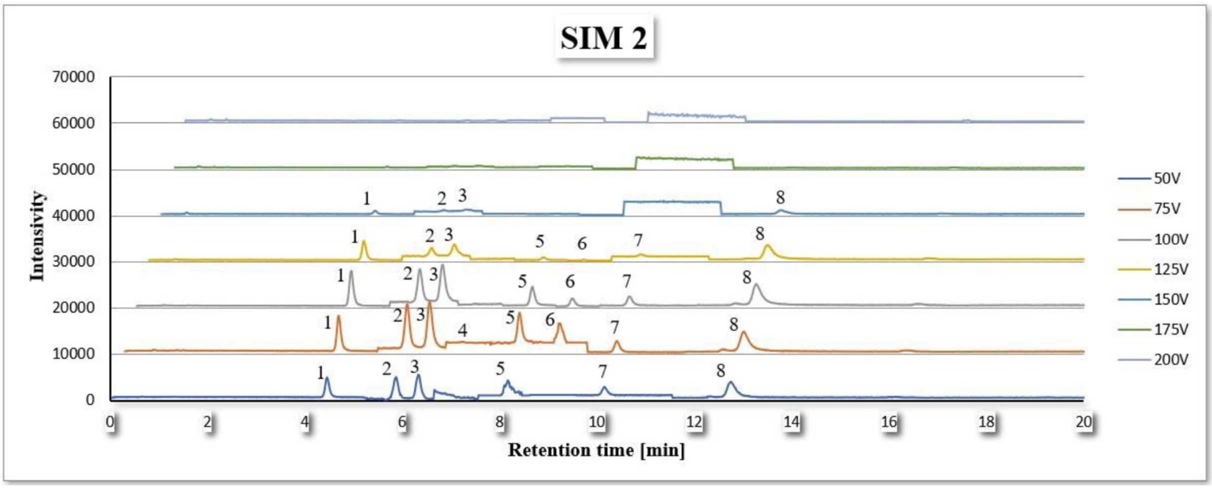

Figure S2. Chromatogram of signal intensity for different CE for SIM 2.

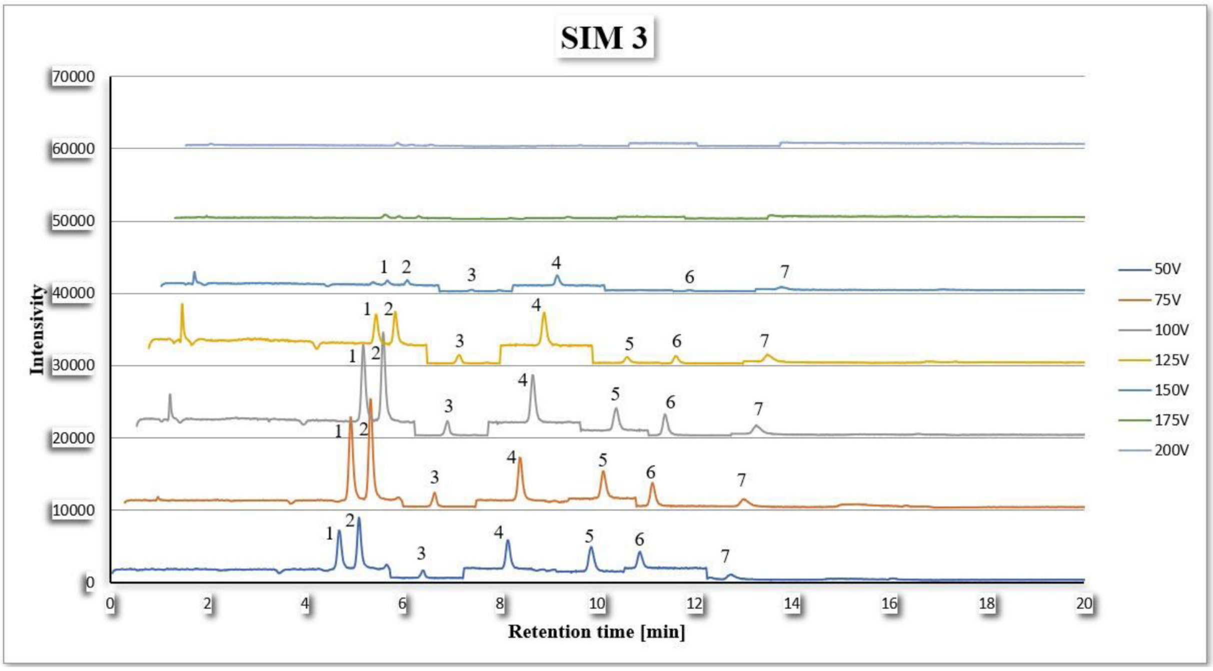

Figure S3. Chromatogram of signal intensivity for different CE for SIM 3.

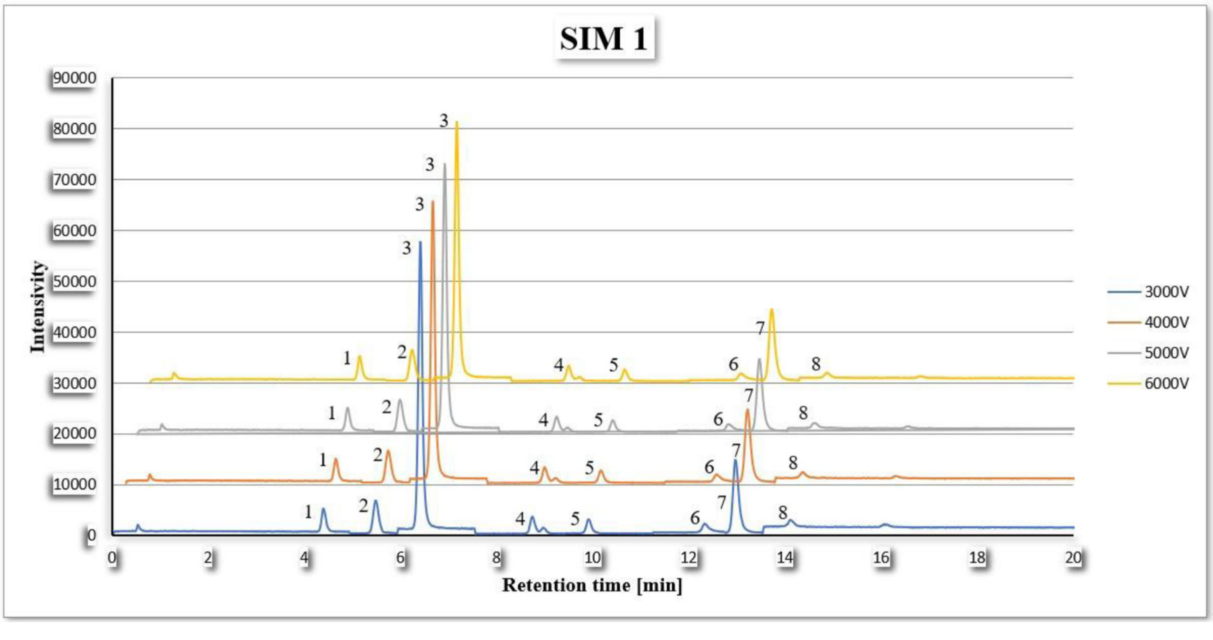

Figure S4. Chromatogram of signal intensivity for different capillary voltage for SIM 1.

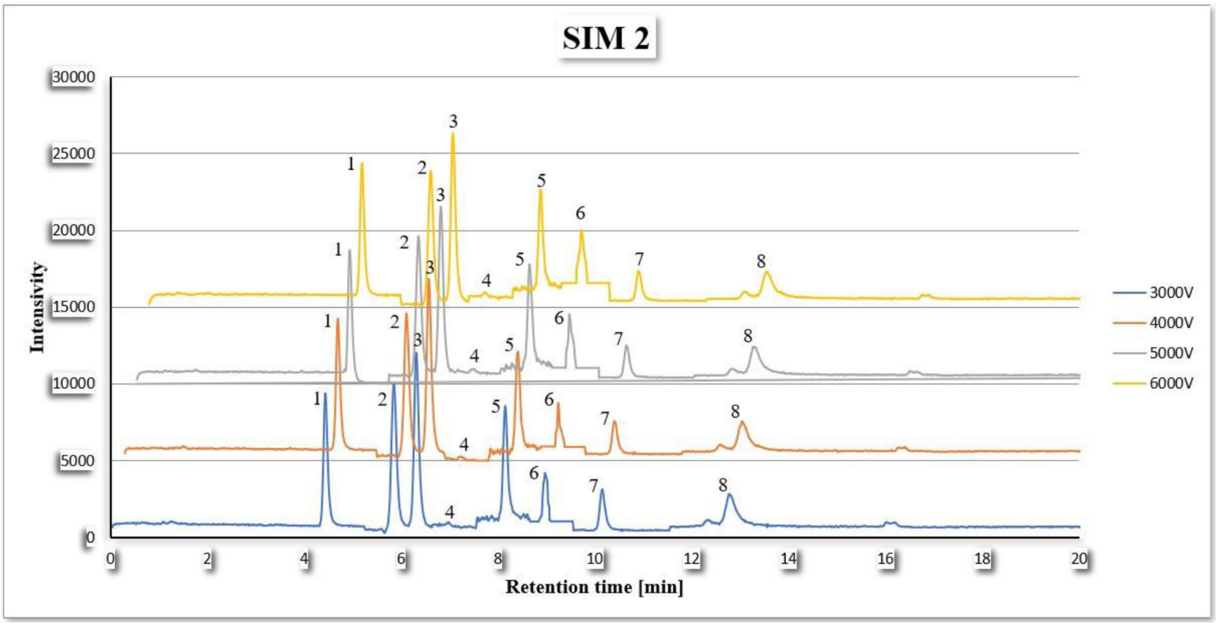

Figure S5. Chromatogram of signal intensivity for different capillary voltage for SIM 2.

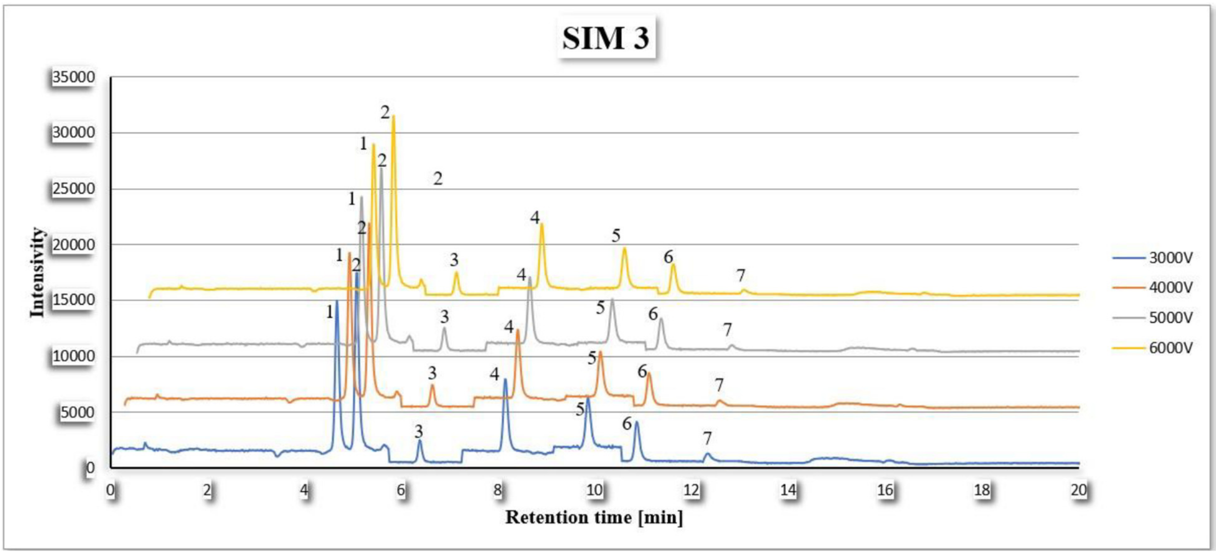

Figure S6. Chromatogram of signal intensivity for different capillary voltage for SIM 3.
